# Interfacial Reaction and Mechanical Properties of Sn-Bi Solder joints

**DOI:** 10.3390/ma10080920

**Published:** 2017-08-09

**Authors:** Fengjiang Wang, Ying Huang, Zhijie Zhang, Chao Yan

**Affiliations:** 1 Key Laboratory of Advanced Welding Technology of Jiangsu Province, Jiangsu University of Science and Technology, Zhenjiang 212003, China; huangy.just@gmail.com (Y.H.); zjzhang@just.edu.cn (Z.Z.); 2 School of Materials Science and Engineering, Jiangsu University of Science and Technology, Zhenjiang 212003, China

**Keywords:** Sn-Bi, solder, intermetallic compound, mechanical property, microstructure, aging

## Abstract

Sn-Bi solder with different Bi content can realize a low-to-medium-to-high soldering process. To obtain the effect of Bi content in Sn-Bi solder on the microstructure of solder, interfacial behaviors in solder joints with Cu and the joints strength, five Sn-Bi solders including Sn-5Bi and Sn-15Bi solid solution, Sn-30Bi and Sn-45Bi hypoeutectic and Sn-58Bi eutectic were selected in this work. The microstructure, interfacial reaction under soldering and subsequent aging and the shear properties of Sn-Bi solder joints were studied. Bi content in Sn-Bi solder had an obvious effect on the microstructure and the distribution of Bi phases. Solid solution Sn-Bi solder was composed of the β-Sn phases embedded with fine Bi particles, while hypoeutectic Sn-Bi solder was composed of the primary β-Sn phases and Sn-Bi eutectic structure from networked Sn and Bi phases, and eutectic Sn-Bi solder was mainly composed of a eutectic structure from short striped Sn and Bi phases. During soldering with Cu, the increase on Bi content in Sn-Bi solder slightly increased the interfacial Cu_6_Sn_5_ intermetallic compound (IMC)thickness, gradually flattened the IMC morphology, and promoted the accumulation of more Bi atoms to interfacial Cu_6_Sn_5_ IMC. During the subsequent aging, the growth rate of the IMC layer at the interface of Sn-Bi solder/Cu rapidly increased from solid solution Sn-Bi solder to hypoeutectic Sn-Bi solder, and then slightly decreased for Sn-58Bi solder joints. The accumulation of Bi atoms at the interface promoted the rapid growth of interfacial Cu_6_Sn_5_ IMC layer in hypoeutectic or eutectic Sn-Bi solder through blocking the formation of Cu_6_Sn_5_ in solder matrix and the transition from Cu_6_Sn_5_ to Cu_3_Sn. Ball shear tests on Sn-Bi as-soldered joints showed that the increase of Bi content in Sn-Bi deteriorated the shear strength of solder joints. The addition of Bi into Sn solder was also inclined to produce brittle morphology with interfacial fracture, which suggests that the addition of Bi increased the shear resistance strength of Sn-Bi solder.

## 1. Introduction

Sn-Ag-Cu Pb-free solder alloy is commonly employed as the interconnected material to replace traditional Sn-Pb solder due to its superior wettability, reliability and mechanical properties [1]. The melting temperature (MP) for Sn-Ag-Cu Pb-free solder ranges from 217 °C to 221 °C and is far higher than 183 °C for Sn-37Pb eutectic solder, which means that a higher temperature will be required during assembling the components with boards. However, with continued shrinkage in size and thickness of electronic packaging, higher soldering temperature from Sn-Ag-Cu solder inevitably results in the thermal warpage on the thin or ultra-thin components [2]. To solve this reliability issue, development of Pb-free solder with MP close to or lower than Sn-37Pb solder is recently pursued in electronic industry [3].

**Figure 1:**
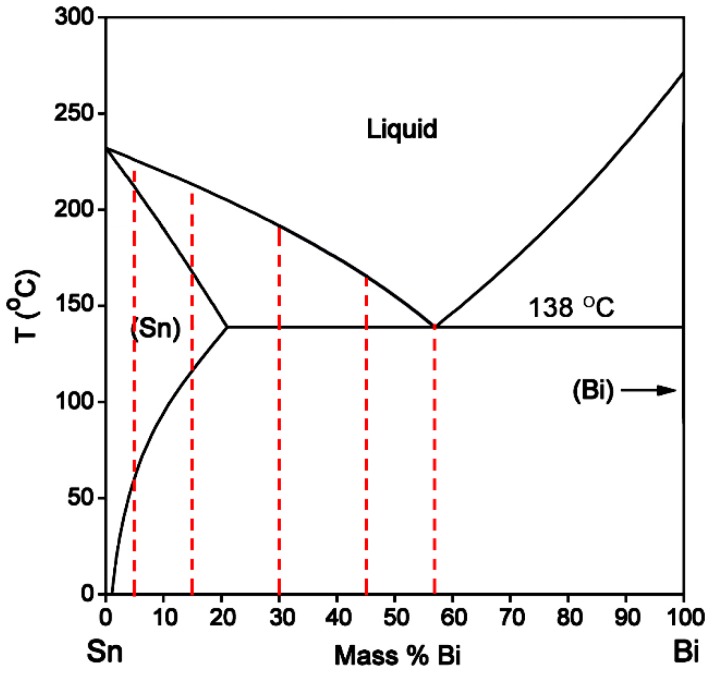
Sn-Bi Phase diagram [10] with dash lines illustrating the concentrations used in this study.

Solder in Sn-Bi binary system provides a low temperature solution. Based on Sn-Bi binary phase diagram shown in Figure 1, with Bi alloyed into Sn, the liquidus temperature of Sn-Bi solder can be continuously decreased from 232 °C at the melting point of Sn to 138 °C at eutectic composition of Sn-58Bi. It can be clearly seen that the solid solution Sn-Bi solder with Bi content under the maximum solubility of Bi in Sn, namely 21 wt %, provides the potential alternative for Sn-37Pb solder, while hypoeutectic-to-eutectic Sn-Bi solder with higher Bi content is helpful to realize lower temperature soldering. Nowadays, many researchers have studied the microstructure and mechanical properties of Sn-Bi solder with different Bi content. Shen et al. [4] investigated the creep performance of Sn-Bi with Bi content of 3%, 10%, 50% and 57% using the nanoindentaiton method and found that the transition stress decreased with Bi content. Lai et al. [5] and Ye et al. [6] studied the hardness and bend fracture energy of Sn-Bi with Bi content of 3.6%, 7.25%, 14.5%, 29% and 58%, and found that Sn-14.5Bi solder had the highest among them. Osório et al. [7] and Silva et al. [8,9] used a directionally solidified method to manufacture Sn-40Bi and Sn-52Bi hypoeutectic solders, and then investigated their microstructure and tensile properties. In summary, Bi addition would increase the mechanical properties of Sn-Bi solder.

Moreover, during soldering with Cu substrate, an intermetallic compound (IMC) layer formed at the solder/Cu interface is necessary for the metallurgical joining. Currently, the non-eutectic Sn-Bi solder for interfacial reaction only includes Sn-5Bi [11] and Sn-10Bi [12,13], where Cu_6_Sn_5_ and Cu_3_Sn IMCs were formed at the interface and the growth kinetics of Cu-Sn IMC layers were studied under the thermal aging, while most research was focused on Sn-58Bi eutectic solder. Lee et al. [14] investigated the interfacial reaction products of Sn-58Bi solder on Cu and electroless nickel-immersion gold (ENIG), and the corresponding IMC were Cu_6_Sn_5_ and Ni_3_Sn_4_, respectively. Hu et al. [15] studied the growth kinetics of Cu-Sn IMC between molten Sn-58Bi and Cu, and found that the IMC thickness followed the linear growth on soldering temperature. Li et al. [16] systemically studied the effect of the third alloying element on IMC growth of molten Sn-58Bi on Cu, and found that only Zn was effective to depress the IMC growth. 

It seems that Bi addition into Sn did not enroll into the interfacial Cu-Sn reaction during the formation of Cu-Sn IMCs. However, in Sn-58Bi/Cu joint, our previous paper [17] and Zou et al. [18] have found that the formation of Bi-rich layer and the segregation of Bi atoms were easily developed at the interface, which would deteriorate the joint strength. Therefore, the Bi existence in Sn-Bi solder will affect the interfacial behaviors occurring in Sn and Cu. It is necessary to study the effect of different Bi addition into Sn on the interfacial reaction on Cu. In this study, the relationship between Bi distribution from different Bi addition in Sn-Bi solder, the interfacial characteristics at the interface and the resulting joint strength were systematically studied. This paper selects Sn-Bi solders with different Bi content ranging from 5–58 wt %, and the effect of Bi content on the microstructure of solders, the interfacial behaviors between solder and Cu during soldering and the following aging conditions, and the shear strength on ball joints are studied.

## 2. Experimental

Sn-Bi solders with different Bi content were manufactured in this study. The detailed compositions include two solid solution compositions of Sn-5Bi and Sn-15Bi, two hypoeutectic compositions of Sn-30Bi and Sn-45Bi, and an eutectic composition of Sn-58Bi. These solder alloys were prepared from pure Sn and Bi. For each solder, the weighted Sn and Bi raw materials were melted in a ceramic crucible at 600 °C for 30 min with protection from the molten salt of 1.3KCl:1LiCl. Mechanical stirring was required to homogenize the solder alloy. The molten solder was cooled to 300 °C, and then chilled and casted with graphite mold.

To prepare the joints for interfacial observation, the wetted samples were prepared with solder globules on Cu substrate. The solder globule with a weight about 0.2 g was manufactured in the molten rosin. Oxygen-free Cu (OFC) with dimensions of 15 mm × 15 mm × 1 mm was used as the substrate. Before wetting, the wetted surface on copper plate was carefully polished to a surface finish with 1 μm using diamond solution, and then ultrasonically cleaned with acetone and distilled water. During wetting, the Sn-Bi solder globule was coated with a commercial rosin-based soldering flux and placed on the Cu plate. The soldering temperature and time was 250 °C and 60 s, respectively. To study the interfacial evolution in solder joints under aging conditions, the wetted samples were then aged in silicone oil with an aging temperature of 135 °C and aging time from 10 to 40 days. The wetted and aged joints were cross-sectioned for interfacial analysis. To prepare the samples for microstructural observation, the as-casted solder alloys, the as-wetted and aged samples were then epoxy-mounted and polished to a 0.05 μm surface finish with colloidal silica. The microstructure in the solder system and the interfacial structure in the joint system were observed by scanning electron microscopy (SEM, ZEISS MERLIN, Germany), and the elemental composition was detected by energy dispersive spectrum (EDS, OXFORD INCA, Germany). The thickness of IMC layer at the interface was determined by dividing the area on the IMC layer by its measured length, and was averaged by five SEM images.

**Figure 2:**
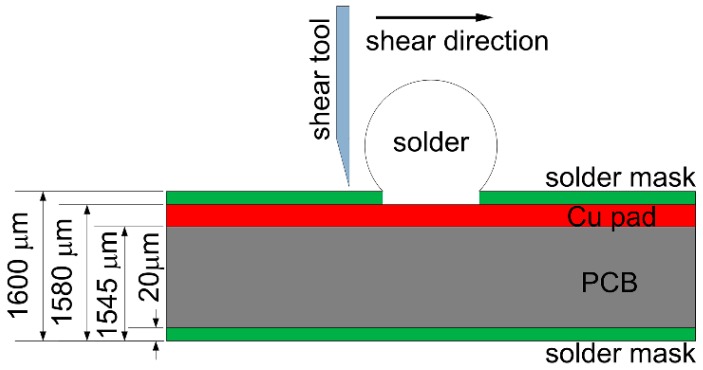
Schematic diagram on the ball shear test.

To evaluate the effect of Bi content on the mechanical properties of Sn-Bi as-soldered joints, a ball shear test was used with the method and the various parameters on printed circuit board (PCB) illustrated in Figure 2. Sn-Bi solder ball with the diameter of 0.76 mm was reflowed on Cu pad with 0.6 mm diameter at the temperature of 250 °C for 60 s. The shear test was finished with the shear height of 20 μm and shear speed of 0.1 mm/s. After shear tests, the fracture surface was observed with SEM, and the element mapping at the fracture morphology was detected by EDS.

## 3. Results and Discussion

### 3.1. Microstructure of Sn-Bi Solder Alloys

**Figure 3:**
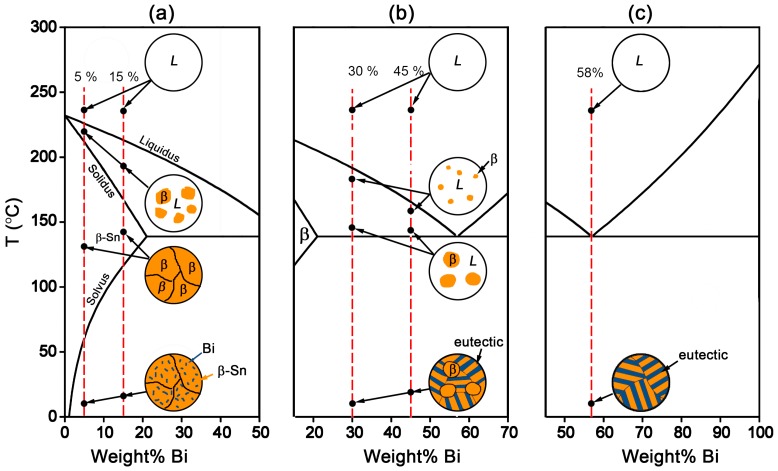
Microstructural evolution in Sn-Bi solder system: (**a**) solid solution Sn-Bi; (**b**) hypoeutectic Sn-Bi and (**c**) eutectic Sn-Bi.

From the Sn-Bi binary phase diagram shown in Figure 1, there exists an eutectic reaction at 138 °C with the composition of Sn-58Bi. β-Sn phase is the solid solution rich in Bi with the solubility limit of 21 wt % occurring at eutectic temperature, while there is almost no solution for Sn in Bi. Accordingly, the microstructural evolution for Sn-Bi solder system is illustrated in Figure 3. Sn-5Bi or Sn-15Bi belongs to the solid solution region. With the temperature reaching the liquidus line, β-Sn solid solution phases will gradually crystallize from the liquid phase until reaching the solidus line. After the solvus line, Bi will precipitate from the solid solution phases, and the final microstructure for the solder is composed of β-Sn solid solution matrix with finely distributed Bi precipitates, as illustrated in Figure 3a. For Sn-30Bi and Sn-45Bi hypoeutectic solder alloys, the Bi composition in Sn-Bi solder is over the solubility limit in the β-Sn, but less than the eutectic composition. After reaching the liquidus line during solidification, the primary β-Sn phases will be formed in the liquid solder. When the solder temperature continually decreases to 138 °C, eutectic reaction occurs in the left liquid phase in order to produce a striped eutectic microstructure with alternating Sn and Bi phases. Therefore, the final microstructure for Sn-30Bi and Sn-45Bi is then composed of the primary β-Sn phases surrounded by stripes of the eutectic phases, as illustrated in Figure 3b. Sn-58Bi is the solder with an eutectic composition, and therefore an eutectic reaction directly occurs to produce the eutectic structure when the temperature of liquid solder reaches 138 °C. The final microstructure of Sn-58Bi solder is fully composed of the striped eutectic structure with alternating β-Sn and Bi phases, as seen in Figure 3c.

**Figure 4:**
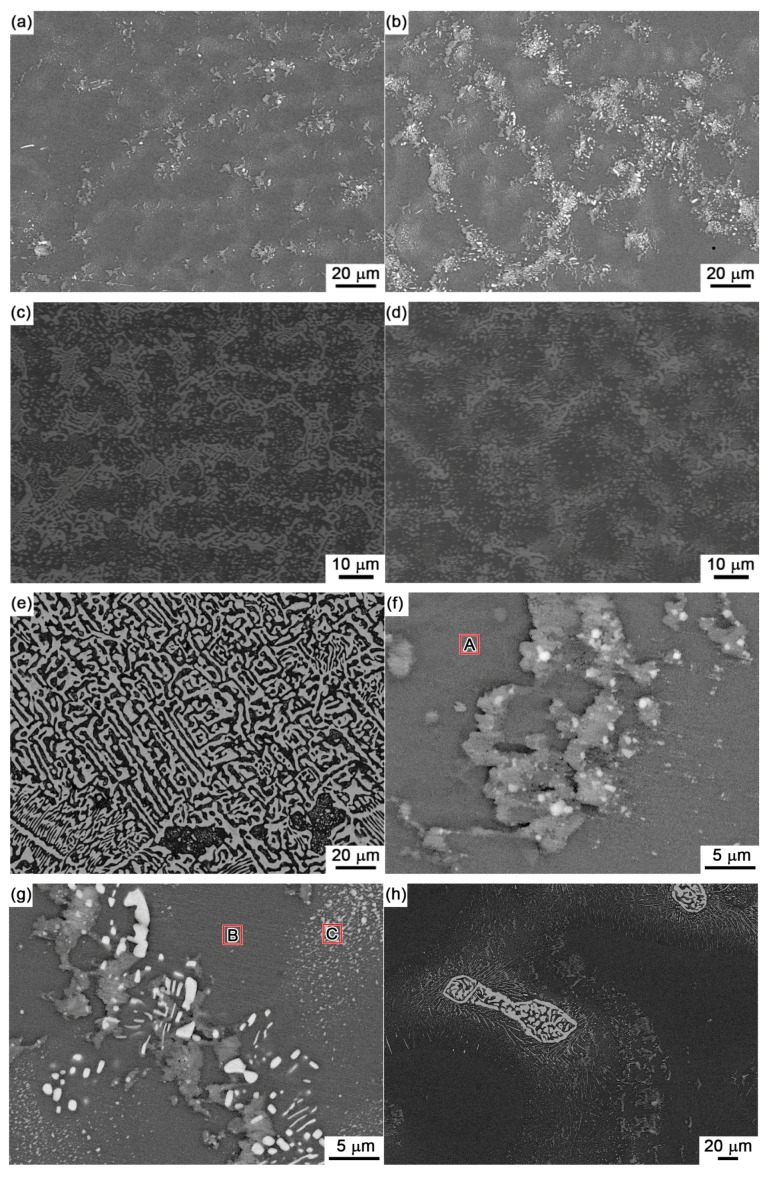
SEM images on the microstructure of Sn-Bi solder alloy: (**a**) Sn-5Bi; (**b**) Sn-15 Bi; (**c**) Sn-30Bi; (**d**) Sn-45Bi; (**e**) Sn-58Bi; (**f**,**g**) higher magnification on Sn-5Bi and Sn-15Bi solder; and (**h**) Sn-15Bi with furnace cooling.

Figure 4 shows the back scattered-electron (BSE) images on the microstructure for Sn-5Bi, Sn-15Bi, Sn-30Bi, Sn-45Bi and Sn-58Bi solder alloys. There are two kinds of phases in them: grey β-Sn phases and white Bi-rich phase. The distribution of Bi phases was obviously changed by Bi content. Meanwhile, the compositions of phases for the regions marked in Figure 4 were also detected by EDS analysis.

As shown in Figure 4a for Sn-5Bi solder, the microstructure was mainly composed of β-Sn grains surrounded by rarely distributed small Bi particles. Bi distribution was magnified with the image shown in Figure 4f. It can be clearly seen that Bi phases were mainly precipitated from the Sn phases. From EDS results on β-Sn phase (region A), there still exists about 3.17 wt % Bi with solid solution in β-Sn after solidification. With Bi content in Sn-Bi increased from 5 wt % to 15 wt %, large amounts of Bi particles were obviously observed both along and within the β-Sn grains. The more greatly magnified BSE image on Sn-15Bi solder is shown in Figure 4g. In region B, the solid solubility of Bi atoms in β-Sn phase was about 5.49 wt % from EDS analysis. Therefore, other Bi atoms were precipitated both along β-Sn phase grains and within β-Sn phase grains with very small sizes such as the region C in Figure 4g, due to the limited solubility of Bi in Sn at room temperature. Therefore, just as shown in Figure 3, the microstructure of Sn-5Bi and Sn-15Bi solid solution solders is composed of the β-Sn matrix embedded with fine Bi particles along or within the β-Sn phase grains. The microstructure in Figure 3 was predicted from the equilibrium crystallization. However, it should be mentioned that the cooling rate within the graphite mold should be much higher than the equilibrium solidification in the present study. There should be some differences between experimental and predicted structures. Therefore, we especially observed the microstructure of Sn-15Bi solder under furnace cooling, as seen in Figure 4h, which was completely different from microstructure in Figure 4b under rapid cooling conditions. Due to the adequate precipitation of Bi atoms from β-Sn, large amounts of bulk Bi phases were observed along β-Sn grains. Because the substrate and the component also provide the cooling channels for Sn-Bi in the solder joints, most Bi atoms will also be kept in β-Sn phases due to the non-equilibrium crystallization.

Figure 4c,d show the BSE microstructure of Sn-30Bi and Sn-45Bi hypoeutectic solders. The dark and bright regions are primary β-Sn phases and Sn-Bi eutectic structure, respectively. As mentioned above, the primary β-Sn phases were firstly solidified from the liquid solder, and then the eutectic reaction occurred on the remaining liquid to form the eutectic structure among them. The increase in Bi content in Sn-Bi hypoeutectic solder promotes the increase of the amount of eutectic structure and a decrease in the primary β-Sn phases. Therefore, the microstructure of Sn-30Bi and Sn-45Bi hypoeutectic solders is composed of the primary β-Sn phases surrounded by the networked Sn-Bi eutectic phases.

Finally, Figure 4e shows the microstructure of Sn-58Bi solder. It is a typical striped eutectic structure with alternated β-Sn phases and Bi phases due to the instantaneous phase separation during solidification. Similarly, the primary β-Sn phases still formed in the right corner of solder microstructure due to the non-equilibrium solidification.

### 3.2. Microstructure of Sn-Bi Solder Joints at the Interface

**Figure 5:**
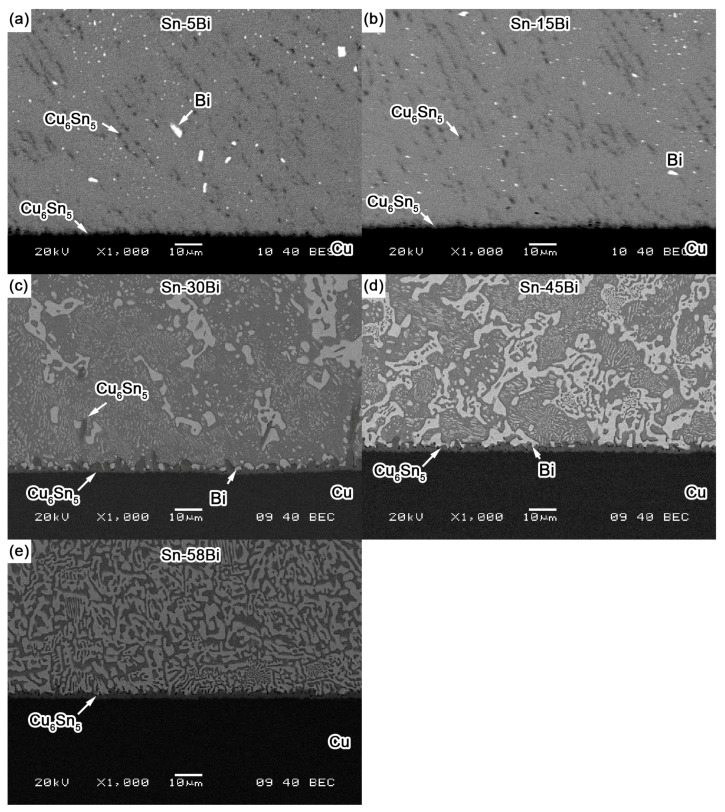
Interfacial structure of Sn-Bi solder joints after soldering: (**a**) Sn-5Bi; (**b**) Sn-15 Bi; (**c**) Sn-30Bi; (**d**) Sn-45Bi; and (**e**) Sn-58Bi.

Figure 5a–e show the BSE images on the interfacial structure in Sn-Bi solder joints after wetting. It is clearly seen that an IMC layer was produced between Sn-Bi solder and Cu substrate. Its composition was confirmed as Cu_6_Sn_5_ by EDS analysis and was independent of the Bi content in the solder. At the interfaces of Sn-5Bi and Sn-15Bi solder shown in Figure 5a,b, the morphology of IMC layer shows a typical scallop type, and the thickness of IMC layer is about 1 μm. Besides the interfacial Cu_6_Sn_5_, it is interesting to find that a huge quantity of Cu_6_Sn_5_ IMC flakes was also produced in the solder matrix, but the amount of these IMC flakes in Sn-15Bi solder was less than that in Sn-5Bi. The precipitated Bi particles from soldering were uniformly distributed within the solder matrix. In Sn-30Bi solder joint shown in Figure 5c, the IMC thickness grew a little more than that in Sn-5Bi or Sn-15Bi solder joints. The scallop-type morphology was also observed for the IMC layer. Different from the interfaces in Sn-5Bi or Sn-15Bi, Bi-rich phase segregation was clearly observed in the grooves between the scallop-type grains. In the solder matrix, Bi atoms existed with both small particles and large bulk phases, while Cu_6_Sn_5_ IMC flakes were seldom observed in the bulk solder compared with Figure 5a or Figure 5b. In Sn-45Bi and Sn-58Bi solder joints, as shown in Figure 5d,e, more Bi-rich phases accumulated at the interface between Sn-Bi solder and the Cu_6_Sn_5_ IMC layer, while no Cu_6_Sn_5_ flakes were observed in the solder matrix. The morphology of the IMC layer also became slightly flattened but thicker. The IMC thickness is about 2 μm.

**Figure 6:**
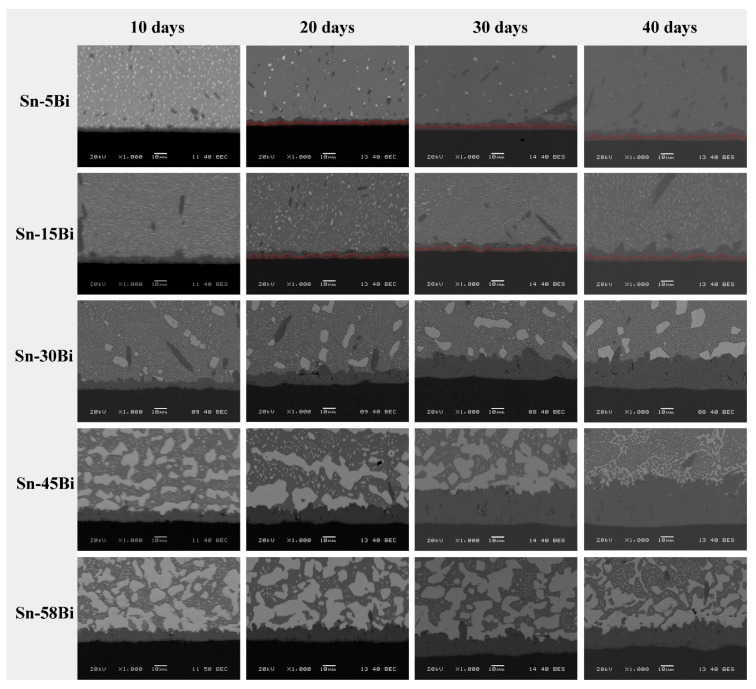
Interfacial evolution of Sn-Bi solder joints with different Bi content under the aging condition.

The effect of aging on the interfacial behaviors of solder joints was then studied. Figure 6 illustrates the representative backscattered electron (BSE) images on Sn-Bi/Cu samples aged at 135 °C with aging duration of 10, 20, 30 and 40 days, respectively. The IMC thickness obviously increased with the aging time. In Sn-5Bi and Sn-15Bi solder joints, with increasing of the IMC thickness at the interface, the Cu_3_Sn IMC layer was produced between Cu_6_Sn_5_ and Cu, which was sketched by the red area in Figure 6, and its thickness also increased with the aging time. In the solder matrix, because some Bi atoms were dissolved into the β-Sn grains during the soldering condition, they were segregated to form small Bi particles during isothermal aging, which were uniformly distributed in the solder matrix as observed in Figure 6. The large amounts of Cu_6_Sn_5_ IMC flakes formed in the soldering condition, as seen in Figure 5a,b, were coarsened and combined with the aging time. In Sn-30Bi and Sn-45Bi hypoeutectic solder joints, the most important observation at the interface is the obvious growth of Cu_6_Sn_5_ layer and the prohibition on the formation of Cu_3_Sn layer between Cu_6_Sn_5_ and Cu. Bi-rich phases were obviously coarsened in the solder matrix and were also accumulated at the interface between solder and IMC layer due to the Sn consumption during the reaction to form Cu_6_Sn_5_. In the Sn-58Bi solder joint, the rapid growth on the Cu_6_Sn_5_ layer and the inhibition on the Cu_3_Sn layer were similarly observed at the interface, but it is interesting to find that the IMC thickness in the Sn-58Bi solder joint was thinner than that in Sn-30Bi and Sn-45Bi solder joints under the same aging time. At the interface between Sn-58Bi solder and IMC layer, the continuous Bi-rich layer always existed during the aging duration.

**Figure 7:**
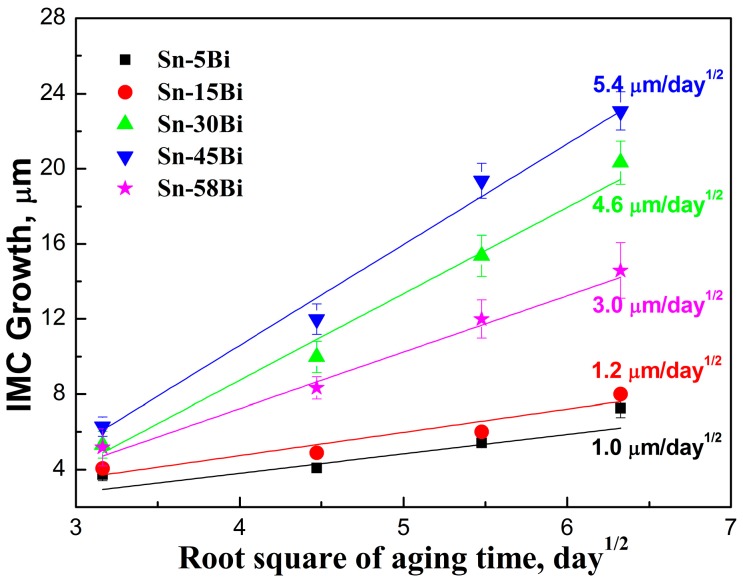
Linear relationship between the thickness of total IMC and the square root of aging days in Sn-Bi solder joints.

It is well known that the IMC growth at the interface in solder joints follows the empirical diffusion formula:

(1) $$X=\sqrt{Dt}+X_{0},$$

where *X* is the total IMC thickness, *X*_0_ is the initial IMC thickness, *t* is the time and *D* is the diffusivity of the IMC layer. Therefore, the growth of IMC thickness (*X* − *X*_0_) was plotted with the square root of aging days (*t*^1/2^) with the results shown in Figure 7. It is clearly seen that Sn-5Bi and Sn-15Bi had the lowest rate of 1.0 μm/day^1/2^ and 1.2 μm/day^1/2^, respectively. With Bi content in Sn-Bi solder increasing to 30 wt % and 45 wt %, the rapid growth on the IMC layer occurred at the interface with the growing rate of 4.6 μm/day^1/2^ and 5.4 μm/day^1/2^, respectively. However, with Bi content in the solder reaching the eutectic composition of Sn-58Bi, the growth rate of the IMC layer at the interface decreased to 3.0 μm/day^1/2^. Therefore, in all of these Sn-Bi solders, Sn-5Bi and Sn-45Bi solder had the lowest and highest growth rate on IMC thickness, respectively.

In the case of the formation of Cu_6_Sn_5_ IMC layer at the interface, it is commonly obtained from the reaction between Sn and Cu atoms with the following reaction:

6Cu + 5Sn → Cu_6_Sn_5_, (2)

where Cu and Sn atoms are from the solder matrix and Cu substrate, respectively. In the case of the formation of the Cu_3_Sn IMC layer at the interface during isothermal aging, it is obtained from the following reactions:

3Cu + Sn → Cu_3_Sn, (3)

9Cu + Cu_6_Sn_5_ → 5Cu_3_Sn, (4)

where the latter reaction plays the dominant role by consuming the Cu_6_Sn_5_ IMC layer. In the case of the formation of Cu_6_Sn_5_ particles or flakes in the solder matrix, it is also obtained from the reaction between Cu and Sn, which means that Cu atoms must diffuse through the Cu-Sn IMC layer into the solder matrix with a longer distance.

**Figure 8:**
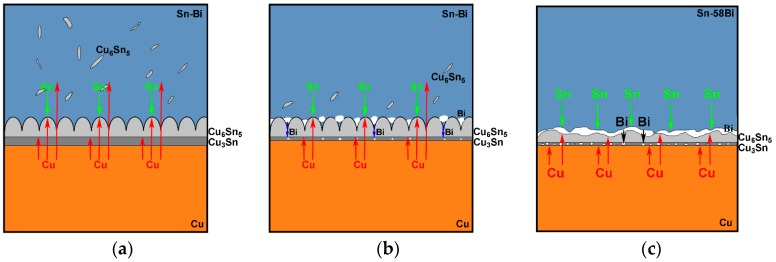
Schematic diagrams on the interfacial behavior in (**a**) Sn-5Bi/Sn-15Bi; (**b**) Sn-30Bi/Sn-40Bi; and (**c**) Sn-58Bi solder joints.

**Figure 9:**
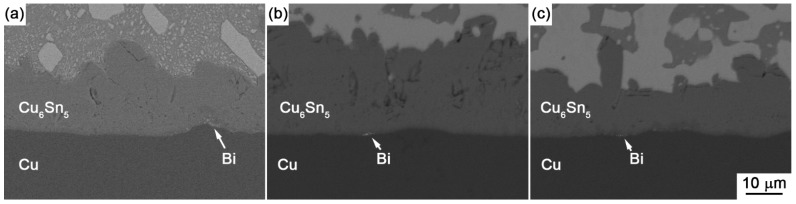
Bi segregation at the interface between Cu_6_Sn_5_ and Cu in (**a**) Sn-30Bi; (**b**) Sn-45Bi; and (**c**) Sn-58Bi aged solder joint with a time of 30 days.

From Figure 7, it can be noted that Bi addition into Sn had the obvious effect on the growth rate of IMC at the interface. In the Sn-Bi/Cu system, Zou et al. [18] verified that Bi atoms were also an important diffusing component to be partly dissolved into Cu_6_Sn_5_ IMC. From Figure 5 and Figure 6, the addition of Bi into Sn solder changed not only the distribution of Cu-Sn IMC in the solder matrix but also the morphology of Cu-Sn IMC at the interface. Therefore, we try to explain the interfacial behaviors from the change on the diffusing fluxes of atoms caused from different Bi addition. The schematic diagrams on the effect of different Bi content in the Sn-Bi solder on the interfacial morphology and IMC growth behavior are illustrated in Figure 8. It should be noted that Cu was the dominant species in Cu-Sn interdiffusion [19]. In Sn-5Bi or Sn-15Bi solder joints illustrated in Figure 8a, a Cu_6_Sn_5_ IMC layer with the scallop-type morphology was produced during soldering. Cu atoms were also diffused from the substrate into solder through the groove between two scallops, and then reacted with Sn atoms to form huge Cu_6_Sn_5_ precipitates within the solder matrix. During isothermal aging, Cu atoms diffused from the substrate not only reacted with Sn atoms diffused from the solder to promote the continuous growth of Cu_6_Sn_5_ IMC layer, but also reacted with the existing Cu_6_Sn_5_ IMC layer to form a Cu_3_Sn layer between Cu_6_Sn_5_ and Cu. For the Cu_6_Sn_5_ IMC layer at the interface, besides the transition to Cu_3_Sn, it was also broken to produce Cu_6_Sn_5_ flakes in the solder matrix close to the interface. The growth on IMC thickness was attributed to them. In Sn-30Bi or Sn-45Bi solder joint, as shown in Figure 8b, Bi particles were precipitated in the grooves of the scallop-type Cu_6_Sn_5_ grains during soldering, which was not observed in Figure 8a because Bi atoms were mainly dissolved in β-Sn phases to produce the solid solution phases for Sn-5Bi or Sn-15Bi. Therefore, the diffusion of Cu atoms into the solder matrix through the grooves of scallops was prohibited. On the other hand, with the growing on interfacial Cu_6_Sn_5_ IMC, more Bi atoms were also accumulated at the interface between solder and the IMC layer due to the depletion of Sn atoms, but were not enough to produce a continuous Bi-rich layer. These enriched Bi phases would inevitably promote the diffusion of Bi atoms from interface into the Cu_6_Sn_5_ phases. During the transition from Cu_6_Sn_5_ to Cu_3_Sn according to Equation (4), the Bi atoms were then precipitated to produce Bi segregation at the interface between Cu_3_Sn and Cu accompanied with the formation of Cu_3_Sn. Figure 9a,b show the enlarged images on the interfaces of Sn-30Bi/Cu and Sn-45Bi/Cu aged joints with 30 days aging, respectively. The Bi segregation is clearly observed between Cu_6_Sn_5_ and Cu, which may be attributed to the reason that the solubility of Bi atoms in Cu_3_Sn was lower than that in Cu_6_Sn_5_ [20]. 

From Figure 6, it is interesting to find that the formation of Cu_3_Sn was very little. The transition from Cu_6_Sn_5_ to Cu_3_Sn was almost depressed at the interface because the dissolution of Bi atoms into IMC possibly increased the driving force on the formation of Cu_3_Sn. On the other hand, Bi precipitation in the grooves of Cu_6_Sn_5_ also inhibited the diffusion of Cu atoms into the solder matrix. Therefore, the diffusing Cu atoms were mainly accumulated at the interface, which promoted the rapid growth of the thickness of the Cu_6_Sn_5_ layer during isothermal aging due to the lack of formation of Cu_3_Sn at the interface and Cu_6_Sn_5_ phases in the solder matrix. Similarly, as illustrated in Figure 8c for Sn-58Bi solder, the difference from Sn-30Bi or Sn-45Bi hypoeutectic solder joint is the formation of a continuous Bi-rich layer close to the interface during isothermal aging. The transition from Cu_6_Sn_5_ to Cu_3_Sn was also depressed by Bi segregation at the interface observed in Figure 9c. The continuous Bi-rich layer partly blocked the interdiffusion of the Cu-Sn couple, and accordingly caused a slower growth rate of IMC at the interface compared with Sn-30Bi or Sn-45Bi hypoeutectic solder joint. In all of these solder joints, another effect on the IMC growth rate is the ratio of aging temperature to the solidus of solder, which is calculated as 0.84, 0.93, 0.99, 0.99 and 0.99 for Sn-5Bi, Sn-15Bi, Sn-30Bi, Sn-45Bi and Sn-58Bi, respectively. It means that there exists a higher diffusion rate of Cu and Sn atoms in Sn-Bi solders with higher Bi content.

### 3.3. Shear Properties on Sn-Bi Solder Joints

**Figure 10:**
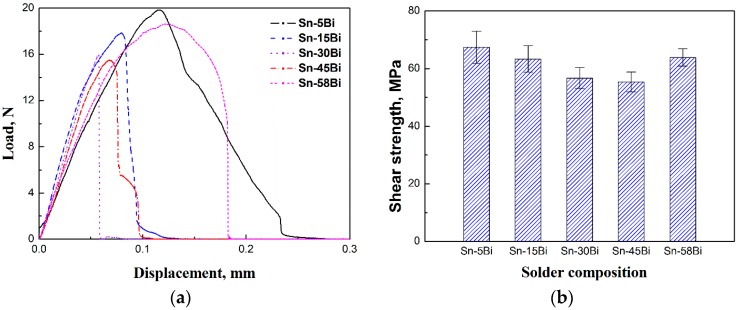
Effect of Bi content in Sn-Bi as-soldered joints on (**a**) load-displacement curves and (**b**) shear strength.

**Figure 11:**
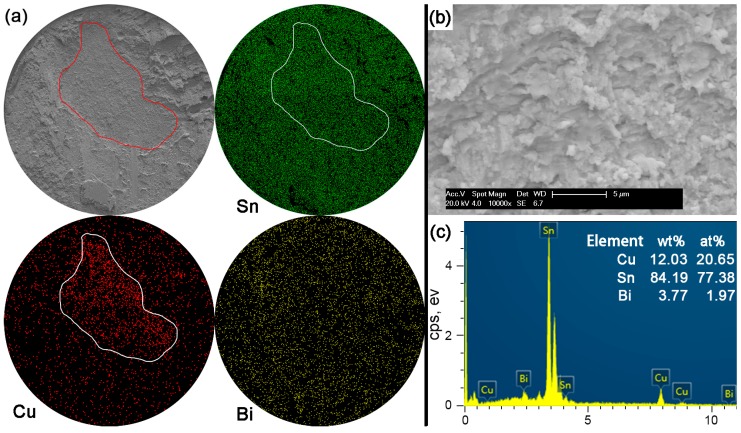
Fracture morphologies of as-soldered Sn-5Bi solder bump: (**a**) SEM image and elemental mapping; (**b**) interfacial fracture at higher magnification; and (**c**) EDS analysis on image (**b**).

**Figure 12:**
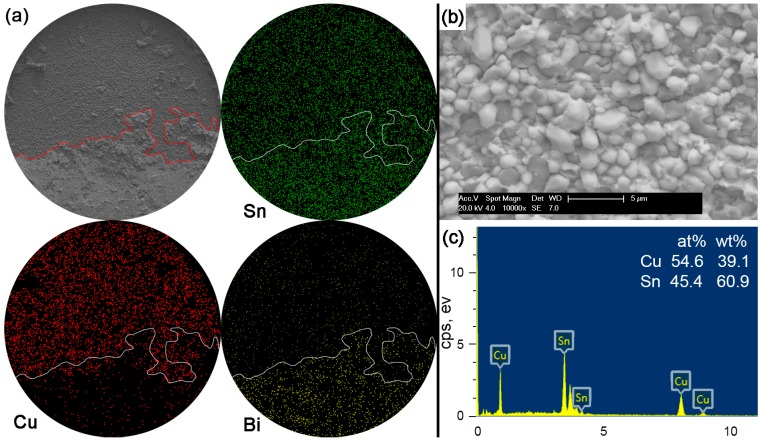
Fracture morphology of as-soldered Sn-15Bi solder bump: (**a**) SEM image and elemental mapping; (**b**) interfacial fracture at higher magnification; and (**c**) EDS analysis on image (**b**).

**Figure 13:**
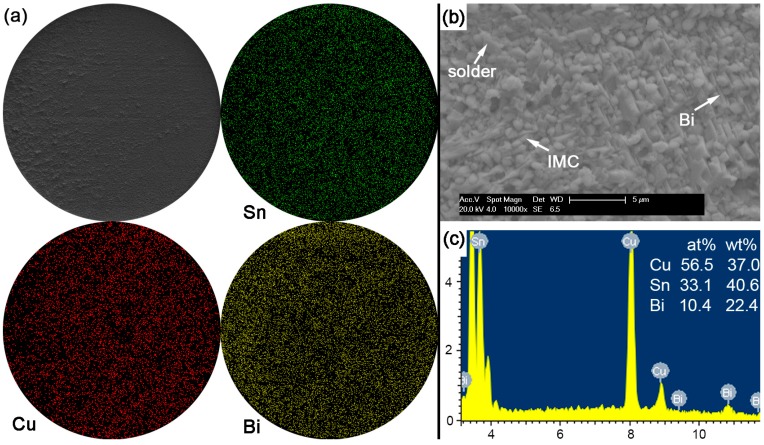
Fracture morphology of as-soldered Sn-30Bi solder bump: (**a**) SEM image and elemental mapping; (**b**) interfacial fracture at higher magnification; and (**c**) EDS analysis on image (**b**).

**Figure 14:**
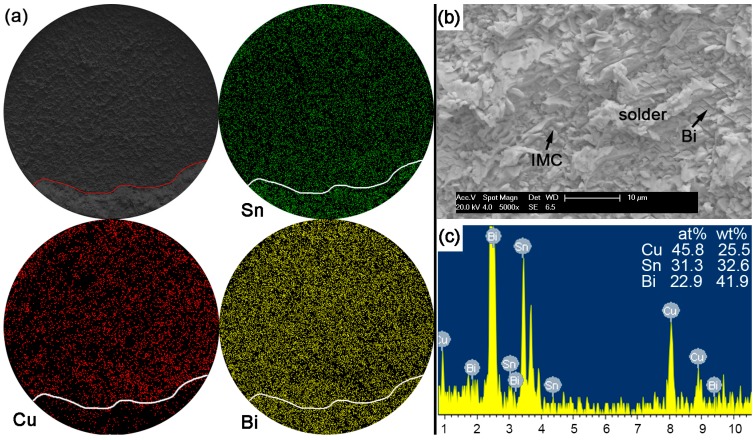
Fracture morphology of as-soldered Sn-45Bi solder bump: (**a**) SEM image and elemental mapping; (**b**) interfacial fracture at higher magnification; and (**c**) EDS analysis on image (**b**).

**Figure 15:**
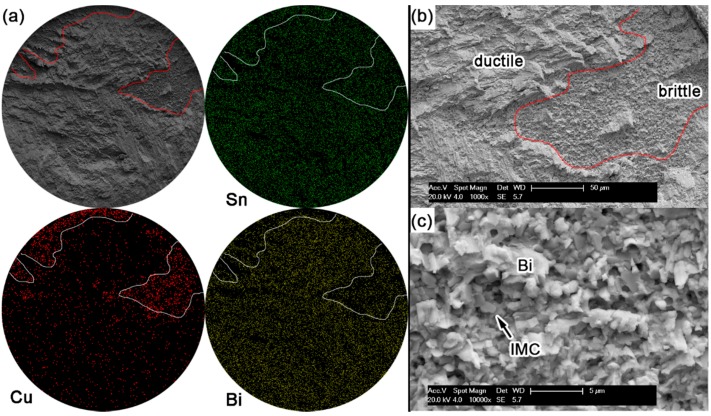
Fracture morphology of as-soldered Sn-58Bi solder bump: (**a**) SEM image and elemental mapping; (**b**) higher magnification on fracture surface with the observation of ductile solder and brittle interface; and (**c**) higher magnification on the brittle area in image (**b**).

Figure 10 shows the displacement-load curves after ball shear tests on Sn-Bi as-soldered joints and the corresponding average shear strength with the effect of Bi content. The shear strength of Sn-Bi solder joints decreased with the increase in Bi content, and then slightly increased for Sn-58Bi eutectic solder. To obtain the effect of Bi content in Sn-Bi solder on the crack propagation and failure mode, the fracture surface morphologies and the corresponding element mapping were detected by EDS, and the results are shown in Figure 11, Figure 12, Figure 13, Figure 14 and Figure 15. The shear direction was always from up to down for all solder joints in these images.

During the ball shear test, because there exists a shear height about 20 μm between the shear tool and the PCB surface, the shear force is always imposed on the bulk solder ball, and the joint strength accordingly reflects the bulk solder strength or the interfacial adhesion strength between solder and Cu substrate. If the shear strength of the bulk solder ball is higher than the interfacial adhesion strength, the fracture propagates along the interface between solder and Cu substrate, which represents a brittle failure mode. Otherwise, ductile failure mode will be observed with the fracture occurring inside the bulk solder ball. Meanwhile, there also exists the quasi-ductile failure mode with more than 50% fracture occurring inside the solder or the quasi-brittle failure mode with more than 50% fracture occurring along the interface, which represents a combination of partial ductile failure mode and partial brittle failure mode. The brittle failure area with fracture propagated along the interface is distinguished with the prominent Cu element mapping from the ductile failure area, and is also marked in Figure 11, Figure 12, Figure 13, Figure 14 and Figure 15. It seems that Bi addition into Sn-based solder, even with 5 wt % Bi content, inevitably produces brittle failure mode, which is completely different from the results on Sn-Ag-Cu Pb-free solder. The ball shear test on Sn-Ag-Cu as-soldered joint always produces the ductile failure inside the bulk solder [21,22]. Their difference is attributed to the reason that Sn-Bi solder exhibits a higher strength but a weaker ductility than Sn-Ag-Cu solder [23] and the Bi element is very prone to embrittlement [6]. As observed from the fracture morphologies, the fracture surfaces almost contain all the above-mentioned failure modes: quasi-ductile mode for Sn-5Bi (Figure 11), quasi-brittle mode for Sn-15Bi (Figure 12), brittle mode for Sn-30Bi and Sn-45Bi (Figure 13 and Figure 14) and ductile to quasi-ductile mode for Sn-58Bi (Figure 15).

In Sn-5Bi solder joints, the performance of bulk solder matrix was obviously improved by both Bi solid solution in the β-Sn phases and large amounts of Cu_6_Sn_5_ flakes produced from the soldering reaction, as observed in Figure 5a. Therefore, during the ball shear test, partial brittle fracture occurred at the interface with the circular area marked in Figure 11a for Sn-5Bi solder bump, which represents the quasi-ductile failure mode because the retained solder area is more than 50%. The circular area was then observed with a 10,000× magnification and the fracture morphology is shown in Figure 11b. EDS analysis was used to confirm the composition at this fracture surface with the result shown in Figure 11c. The interfacial fractured area still contains much Sn-Bi solder with large amounts of Sn and a small amount of Bi besides the Cu atoms from Cu_6_Sn_5_.

Figure 12 shows the fracture morphologies, elemental mapping and the EDS result on the interfacial fracture area for Sn-15Bi solder joints. Compared with the Sn-5Bi solder bump, a more brittle result occurred along the interface to reach a quasi-brittle failure mode with the interfacial fracture area more than 50%. Similarly, the brittle fracture was also observed with a higher magnification with the morphology shown in Figure 12b. It is a typical scallop-type IMC exposed fracture morphology with very little retained solder. The composition of scallops was confirmed as the Cu_6_Sn_5_ phase by the EDS spectrum in Figure 12c. Therefore, although both of the solder alloys were solid solution compositions, more Bi content in Sn-15Bi solder further increased the solder strength and resulted in more fractures along the interface instead of inside the bulk solder and less retained solder on the IMC exposed area compared with Sn-5Bi solder. 

With Bi content in Sn-based solder increased to 30% or 45% in order to constitute the Sn-Bi hypoeutectic solder alloy, the fracture of solder joints in Figure 13 or Figure 14 completely occurred along the interface between solder and Cu substrate with a full brittle failure mode, although there still existed a small retained solder area in Figure 14a for the Sn-45Bi solder joint. From the elemental mapping on fracture morphologies of Sn-30Bi and Sn-45Bi, it is interesting to find that the whole fracture was covered with the uniform distribution of Bi atoms besides the Cu atoms and Sn atoms from Cu_6_Sn_5_. Figure 13b,c and Figure 14b,c show the magnified observation on the brittle fracture area and the corresponding compositional analysis in Sn-30Bi and Sn-45Bi, respectively. In contrast to Sn-5Bi or Sn-15Bi solder bump, the solder residues and cleavage fracture from coarsened Bi phases were easily observed with the amounts increased with Bi content, which came from the Bi precipitation in the grooves or near the interface in Figure 5c,d.

Figure 15 shows the elemental mapping on Sn-58Bi eutectic solder bump and the enlarged fracture morphologies. Most fractures occurred inside the solder. From the fracture morphology on the Sn-58Bi solder joint in Figure 15a, most fractures occurred inside the bulk solder ball, which illustrates a ductile or ductile-to brittle fracture mode. With a highly magnified observation on the interfacial brittle area shown in Figure 15c, small Bi particles were uniformly distributed on the IMC layer. 

The shear strength of Sn-Bi solder bumps should be decided by the performances of solder and the fracture modes during ball shear tests. Ye et al. [6] studied the effect of Bi content in Sn-Bi solder on the mechanical performance of bulk solder alloys, and found that the hardness of Sn-Bi solder reached the highest with Bi content at 7–15 wt %, and then decreased with continuous increasing on Bi content in Sn-Bi solder. Therefore, Sn-5Bi and Sn-15Bi solder bumps show a higher shear strength in Figure 10, and the fracture mode also transits from quasi-ductile to quasi-brittle with the cracks mainly occurring inside the solder bump to the interface between the solder and IMC layer. However, it is interesting to discover that the fractures for Sn-30Bi and Sn-45Bi solder bumps fully occurred at the interface because they had a lower strength than Sn-5Bi and Sn-15Bi solder bumps. As shown in Figure 8b, large amounts of Bi elements were segregated at the interface between the bulk solder and Cu_6_Sn_5_ IMC layer. In Figure 13b and Figure 14b, we also observed the existence of Bi cleavage at the fracture surfaces. It seems that the degradation on Sn-30Bi and Sn-45Bi solder joints were mainly attributed to Bi segregation at the interface. The brittle fracture mode occurring along the interface produces a lower shear strength on Sn-30Bi and Sn-45Bi solder bumps. Sn-58Bi solder had the lowest strength out of all of the Sn-Bi solder alloys in this study; accordingly, the fracture mainly occurred inside the solder bump because the shear strength of solder bump was lower than the interfacial strength, but we still observed the existence of Bi segregation at the interface from the small brittle area in Figure 15c. Sn-58Bi solder joint also shows a slightly higher shear strength due to its ductile fracture mode.

## 4. Conclusions

Sn-Bi solders with different Bi content can realize a low-to-medium-to-high soldering process with the melting temperature ranging from 138 °C to 232 °C. The addition of Bi into Sn changed not only the melting point of solder but also the microstructure, interfacial behaviors in solder with Cu and joint strength. To obtain the effect of Bi content in Sn-Bi solder on them, this study selected five Sn-Bi solders with Bi content ranging from 5–58 wt % including Sn-5Bi and Sn-15Bi solid solution compositions, Sn-30Bi and Sn-45Bi hypoeutectic compositions and Sn-58Bi eutectic composition. The microstructure, interfacial reaction under soldering and subsequent aging and the shear properties of Sn-Bi solder joints were studied, and the relationship between them was discussed. The following results can be concluded:

(1) The Bi content in Sn-Bi solder had an obvious effect on the microstructure and the distribution of Bi phases. Solid solution Sn-Bi solder was composed of the β-Sn phases embedded with fine Bi particles; hypoeutectic Sn-Bi solder was composed of the primary β-Sn phases and Sn-Bi eutectic structure from networked Sn and Bi phases; and eutectic Sn-Bi solder was mainly composed of eutectic structure from short striped Sn and Bi phases.
(2) During soldering with Cu, Bi content in Sn-Bi solder had an obvious effect on the interfacial behavior of solder joints. In the solder matrix of joints, large amounts of fine Cu_6_Sn_5_ flakes were produced with Bi particles in solid solution Sn-5Bi or Sn-15Bi, while they were almost depressed in hypoeutectic Sn-30Bi or Sn-45Bi and eutectic Sn-58Bi. At the interface of solder/Cu, the increase in Bi content slightly increased the interfacial Cu_6_Sn_5_ IMC thickness, gradually flattened the IMC morphology, and promoted the accumulation of more Bi atoms to interfacial Cu_6_Sn_5_.
(3) During the subsequent aging, the growth rate of the IMC layer at the interface of Sn-Bi solder/Cu rapidly increased from solid solution Sn-Bi solder to hypoeutectic Sn-Bi solder, and then slightly decreased for Sn-58Bi solder joints. The continuous formation of Cu_6_Sn_5_ in the solder matrix partly counteracted the growth of interfacial IMC in solid solution Sn-Bi solder joints, while the accumulation of Bi atoms at the interface promoted the rapid growth of the interfacial Cu_6_Sn_5_ IMC layer in hypoeutectic or eutectic Sn-Bi solder through blocking the formation of Cu_6_Sn_5_ in solder matrix and the transition from Cu_6_Sn_5_ to Cu_3_Sn.
(4) Ball shear tests on Sn-Bi as-soldered joints showed that the increase of Bi content in Sn-Bi deteriorated the shear strength of solder joints. The addition of Bi into Sn solder was also inclined to produce brittle morphology with interfacial fracture, which suggests that the addition of Bi increased the shear resistance strength of Sn-Bi solder.
